# Diversity of group A rotavirus on a UK pig farm

**DOI:** 10.1016/j.vetmic.2015.09.009

**Published:** 2015-11-18

**Authors:** Rebecca Chandler-Bostock, Laura R. Hancox, Helen Payne, Miren Iturriza-Gomara, Janet M. Daly, Kenneth H. Mellits

**Affiliations:** a School of Biosciences, Division of Food Science, University of Nottingham, Sutton Bonington LE12 5RD, UK; b Institute of Infection and Global Health, University of Liverpool, L69 7BE, UK; c School of Veterinary Medicine and Science, University of Nottingham, Sutton Bonington, LE12 5RD, UK

**Keywords:** Phylogenetic analysis, Porcine, Group A rotavirus, VP7, VP4

## Abstract

Group A rotaviruses (GARV) are a significant cause of enteritis in young pigs. The aim of this study was to extend our understanding of the molecular epidemiology of porcine GARV in the UK by investigating the genetic diversity of GARV on a conventional farrow-to-finish farm. Faecal samples were obtained from six batches of pigs in 2009 and 8 batches in 2010, when the pigs were 2, 3 (time point omitted in 2009), 4, 5, 6 and 8 weeks of age. Presence of rotavirus was detected by reverse transcriptase-polymerase chain reaction (RT-PCR) in 89% and 80% of samples from 2009 and 2010, respectively. A combination of multiplex PCRs and sequencing identified four VP7 genotypes (G2, G3, G4 and G5) and three VP4 genotypes (P[6], P[7] and P[32]) present in almost every combination over the 2 years. The predominant genotype combination was G5P[32] in 2009 and G4P[32] in 2010. Conservation among the P[32] sequences between 2009 and 2010 suggests that reassortment may have led to the different genotype combinations. There were significant changes in the predominant VP7 genotype prior to weaning at 4 weeks, and post weaning when pigs were moved to a different building. Phylogenetic analysis indicated that introduction of new viruses onto the farm was limited. Taken together, these findings suggest that genetically diverse GARV strains persist within the farm environment.

## Highlights

• Porcine rotavirus A genotypes are highly diverse and persist within a farm.
• Predominant VP7 genotypes change between years and between pre- and post-weaning.
• VP4 genotypes were more conserved than VP7 with some evidence for reassortment.

## Graphical abstract

**Figure:**
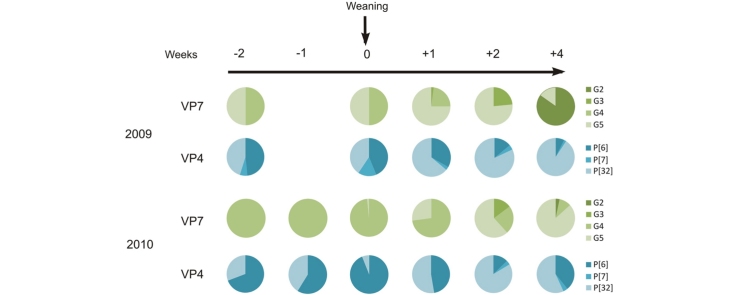

## 1 Introduction

Rotavirus is a major cause of viral gastroenteritis in pigs worldwide. It has a significant economic impact on pig production as a result of the morbidity and mortality caused. Neonatal and weaned pigs have the highest incidence of rotavirus disease (Fu and Hampson, 1987, Linares et al., 2009). Large amounts of virus are shed during rotavirus infection and the virus is highly transmissible (Fu and Hampson, 1989). In a study of natural rotavirus transmission, once piglets shed rotavirus the whole litter was infected within 4 to 10 days (Fu and Hampson, 1989).

Rotaviruses are classified into eight groups (A–H) based on antigenic relationships of VP6, one of the six structural virus proteins (Matthijnssens et al., 2012). Rotaviruses of groups A, B and C can infect pigs, but group A rotaviruses (GARV) are considered the most important due to their high prevalence and pathogenicity. However, emerging group C rotaviruses have caused significant disease in pigs, as well as being often detected in asymptomatic pigs (Collins et al., 2008, Jeong et al., 2015, Marthaler et al., 2013). In addition to the economic importance of GARV in pigs, there is potential for zoonotic transmission to humans (Martella et al., 2006, Steyer et al., 2008 Zhou et al., 2015). Rotaviruses are classified into G and P types based on differences in the outer capsid proteins VP7 (a glycoprotein) and VP4 (a protease-sensitive protein), respectively. These proteins contain epitopes that induce neutralising antibodies. Within GARV, at least 27 G and 37 P genotypes have been identified (Matthijnssens et al., 2011, Trojnar et al., 2013). In addition to antigenic drift arising from the accumulation of point mutations, variation can arise due to reassortment of gene segments if the host cells are co-infected with different viral genotypes.

Knowledge of the molecular epidemiology of porcine GARV is critical for the development of effective prophylactic approaches including vaccines. Twelve G genotypes (G1 to G6, G8 to G12, and G26) and 13 P genotypes (P[1], P[5] to P[8], P[11], P[13], P[19], P[23], P[26], P[27], P[32], and P[34]) have been associated with pigs (Collins et al., 2010a, Collins et al., 2010b, Martella et al., 2007, Matthijnssens et al., 2011, Steyer et al., 2007). In a study of GARV genotypes circulating in UK pigs, six G types (VP7); G2, G3, G4, G5, G9 and G11 and six P types (VP4); P[6], P[7], P[8], P[13], P[23], and P[32] were identified (Chandler-Bostock et al., 2014). A study in Canada concluded that up to four strains of GARV (based on G and P sequences) are present on most pig farms (Lachapelle et al., 2014). The aim of this study was to characterise the genetic diversity of GARV in pigs on an individual UK farm before and after weaning.

## 2 Methods

### 2.1 Sample collection

Samples were collected in two periods commencing in August 2009 and 2010 from a farrow-to-finish farm with approximately 160 sows in the East Midlands, UK. The farm had a continuous flow of pigs through each building with pens cleaned and disinfected on an individual basis. Sows at the same stage of gestation were housed in groups in a dry sow house, moved to a specialised farrowing house two weeks before farrowing then to individual pens early in the farrowing week. The pigs were weaned at 4 weeks of age when they were moved from the farrowing room to the weaner pens and mixed with pigs weaned in the same weekly batch. Samples were collected from a group of six litters from sows farrowing in the same week in 2009 and eight litters in 2010. Ten freshly voided faecal samples were collected from floor when the pigs were 2, 3, 4, 5, 6 and 8 weeks of age (this corresponds to −2, −1, 0, +1, +2 and +4 weeks relative to weaning), with the exception that no samples were taken at 3 weeks of age in 2009. Additional samples were obtained from pigs in the same age range on three visits to the farm at 3-week intervals in July/August 2014. Samples were stored at 4 °C and were processed within 24 h of collection. All samples were obtained and analysed in accordance with the University of Nottingham ethical guidelines.

### 2.2 RNA extraction and RT-PCR amplification of VP7 and VP4

Viral RNA was extracted from faecal samples and VP7 and VP4 rotavirus genes were amplified by RT-PCR as described in Chandler-Bostock et al. (2014).

### 2.3 Genotyping PCR

Porcine-specific multiplex PCRs for VP7 genotypes G2, G4 and G5 and VP4 genotypes P[7] and P[32] were developed for this study. Briefly, 2 μl of product from the RT-PCR amplification of VP7 or VP4 was added to a Taq DNA polymerase PCR mix containing 0.5 pmole/μl of generic VP7 or VP4 reverse primers and genotype-specific forward primers designed to amplify different-sized PCR products. VP7 primers were G2: TCAATTCAACTAGTGAG (681 nucleotide fragment), G4: ATGAATATTCNAATATTNTAGA (529 nucleotides), G5: GATGAAATATGATGCAAA (468 nucleotides). VP4 primers were P[6]: TGTTGATTAGTTGGATTCAA as previously described (Gray and Iturriza-Gomara, 2011), P[7]: GAGCTCAAGTTAATGAGG (624 nucleotides) and P[32]: GTGCTCAAGAAAATNTATG (517 nucleotides). Samples that were RT-PCR positive but could not be assigned a genotype by PCR were sequenced as described in Chandler-Bostock et al. (2014). This combination of multiplex PCR with selective sequencing provided an efficient method to determine the genotypes of the large number of samples obtained.

### 2.4 Phylogenetic analysis

A selection of the RT-PCR positive faecal samples (chosen to represent each group of pigs, each time point and the different genotypes identified) and additional samples obtained from the study farm in 2014 were sequenced, as described in Chandler-Bostock et al. (2014). Sequences have been submitted to the GenBank database (accession numbers VP4: KR261953 – KR261989 and VP7: KR261990 – KR262060). The sequences were aligned with sequences from a UK-wide surveillance study (Chandler-Bostock et al., 2014) and maximum likelihood trees generated using ClustalW and Mega6.

### 2.5 Statistical analysis

Multinomial regression was used to determine whether there were significant differences (*P* ≤ 0.05) in the distribution of genotypes in samples collected at different times pre- and post-weaning (Genstat).

## 3 Results

### 3.1 Rotavirus genotypes identified in 2009 and 2010

In total, 300 samples were collected in 2009, of which 268 (89%) were RT-PCR positive for at least one of the genes amplified (VP7 and VP4). In order of frequency, G5, G2, G4 and G3 VP7 genotypes were identified (Table 1a). The most common VP4 genotype was P[32], followed by P[6] then P[7]. The most common genotype combination was G5P[32] (24%).

Of the 480 samples collected in 2010, 385 (80%) were RT-PCR positive. The same genotypes were present as in 2010 (Table 1b), but the most common genotype combination was G4[P32]. The frequency of P[6] dropped from a quarter of the VP4 genotypes in 2009, to 1.6% in 2010 and there were ten-fold more P[7] samples in 2010 than in 2009. The G2 VP7 genotype represented only 0.2% of samples genotyped in 2010 compared with 16.0% of the samples in 2009.

### 3.2 Rotavirus genotypes in pre- and post-weaning samples

**Fig. 1:**
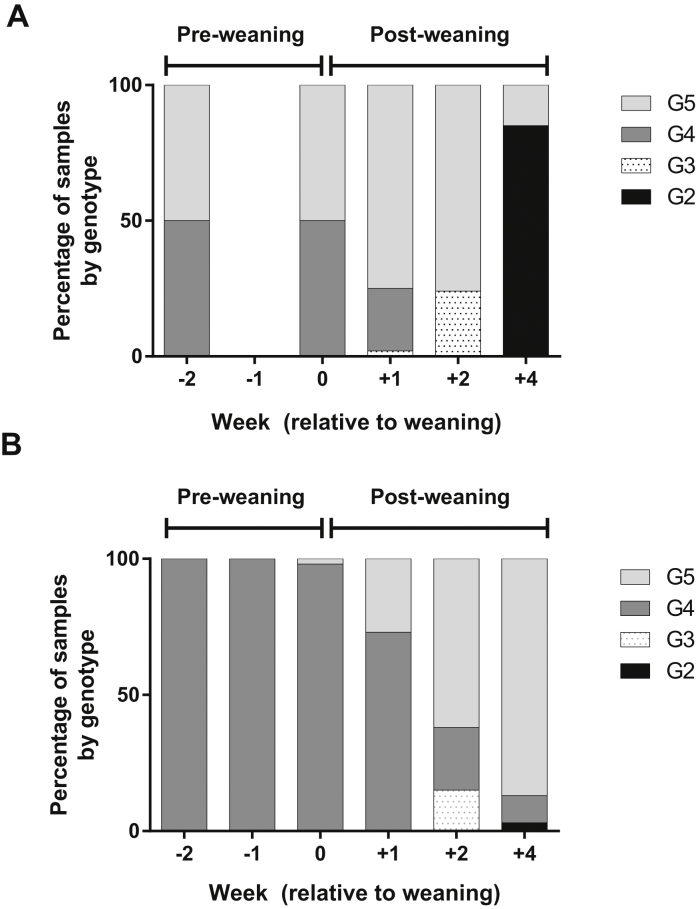
Rotavirus VP7 genotypes present at different time-points (−2, −1, 0, +1, +2 and +4) on the study farm in (A) 2009 and (B) 2010. The proportion of each genotype is shown as a percentage within the bar. ND = no data.

Significant associations (*P* ≤ 0.001) between genotype and age of pigs were found. In 2009, when G5 was the most common VP7 genotype overall, equal proportions of G4 and G5 genotypes were found up to weaning age but no G4 rotaviruses were identified by 2 weeks post-weaning (Fig. 1). G3 genotypes were only detected in post-weaning samples at 5 and 6 weeks of age (+1 and +2 weeks post-weaning), and 80% of the samples were G2 at one month post-weaning. G5 was present at each time point, but only represented 20% of the samples from 8-week-old pigs.

In 2010, when G4 was the most common genotype overall, it represented the majority genotype (>95% of samples) up to weaning but declined to 10% of the samples at 4 weeks post-weaning. At weaning, G5 samples appeared and represented a linearly increasing proportion of genotyped samples up to 87% at 4 weeks post weaning (Fig. 1). Similarly to 2009, G3 samples were present only at 2 weeks post-weaning in 2010 and G2 samples were only detected at 4 weeks post-weaning, but in 2010 only represented 3% of the genotyped samples rather than the majority at this time point.

**Fig. 2:**
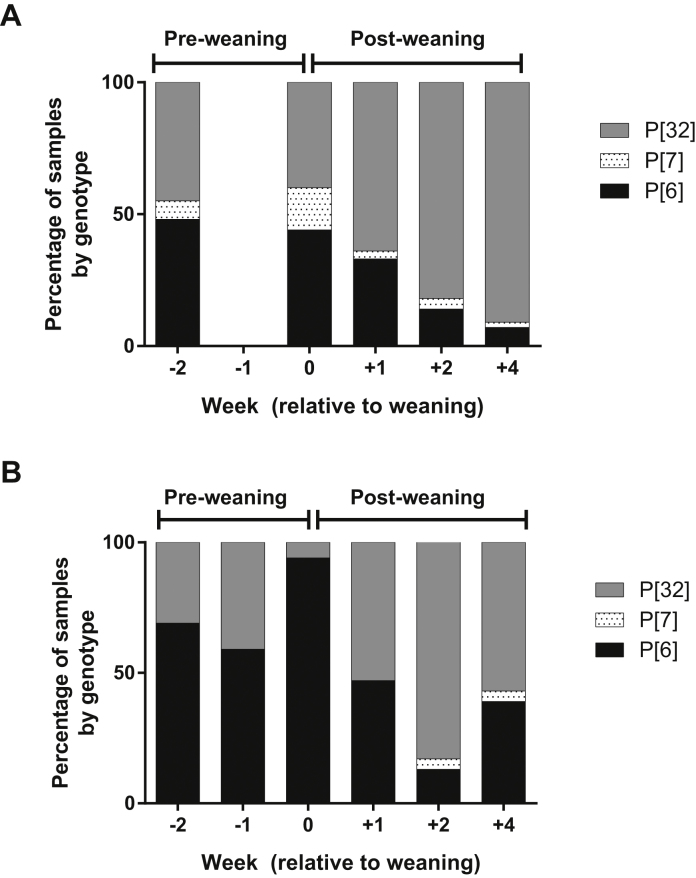
Rotavirus VP4 genotypes present at different time-points (−2, −1, 0, +1, +2 and +4) on the study farm in (A) 2009 and (B) 2010. The proportion of each genotype is shown as a percentage within the bar. ND = no data.

The VP4 genotypes did not change as dramatically over time as the VP7 genotypes (Fig. 2). In 2009, all three of the VP4 genotypes detected were present at each sampling time, but the proportions changed significantly (*P* ≤ 0.05), with the proportion of P[6] samples decreasing over time, while the proportion of P[32] increased. The P[7] genotype was present in a minority of samples at each time point. In 2010, the proportion of the VP4 genotype fluctuated, but the changes were not significantly associated with sample time point. P[7] again comprised the minority of samples and was only present at 2 and 4 weeks post-weaning.

### 3.3 Phylogenetic analysis of rotavirus sequences

**Fig. 3:**
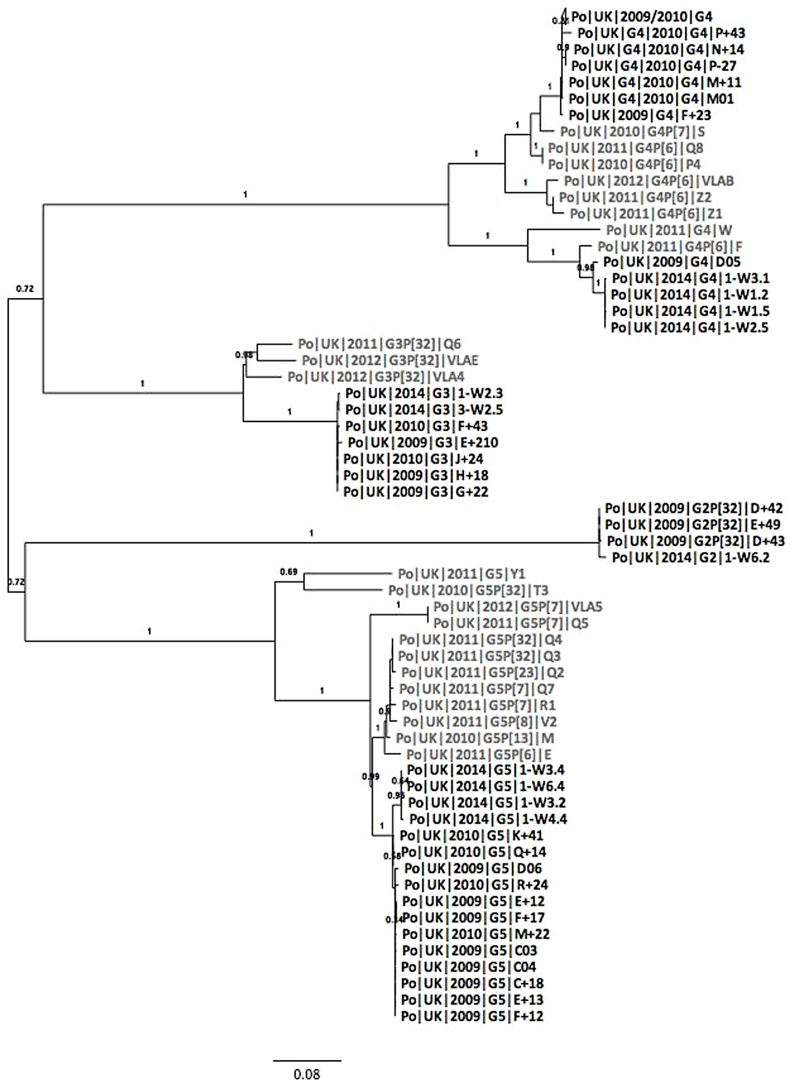

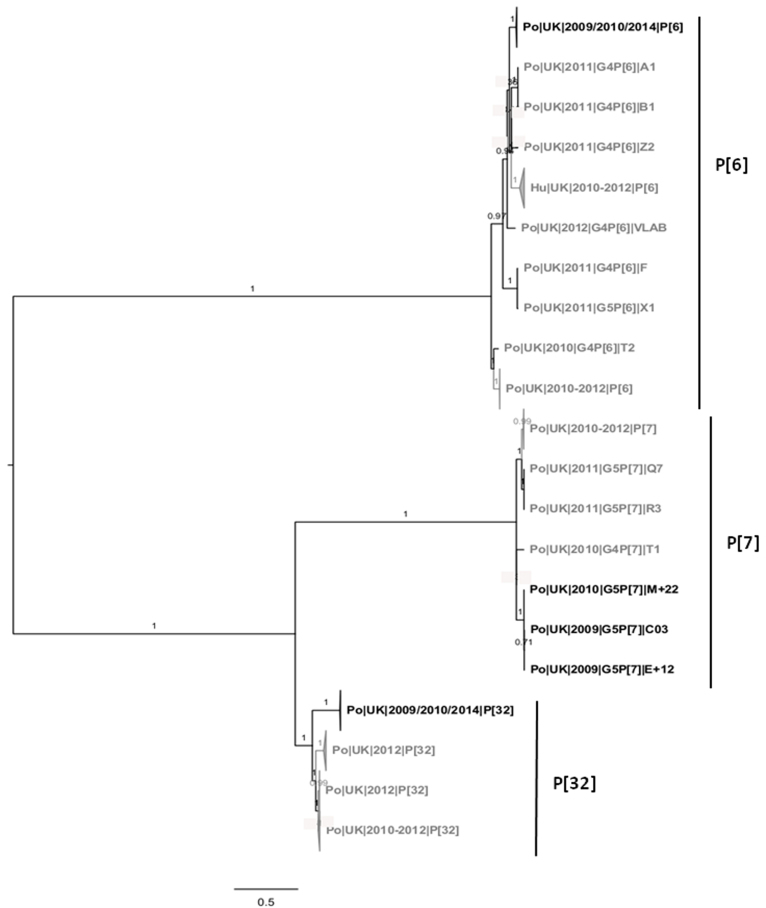
Maximum likelihood trees of (a) VP7 and (b) VP4 nucleotide sequences collected from the same farm (shown in black) and sequences collected in a UK-wide surveillance study (shown in grey) described in Chandler-Bostock et al. (2014). Sequences are denoted as species of origin|country|year collected|genotype|sample ID. Where there are more than 4 identical sequences, braches were collapsed. The bar at the bottom represents nucleotide distance.

Phylogenetic analysis of the VP7 sequences showed that the four G2 sequences obtained from the farm (in 2009 and 2014) were similar to each other (Fig. 3a). The G3 sequences obtained from the farm formed a separate clade to those obtained elsewhere in the UK (Chandler-Bostock et al., 2014). One G4 sequence obtained from the study farm in 2009 grouped with G4 sequences obtained from the same farm in 2014 whereas the rest of the G4 sequences clustered together with G4 sequences obtained from elsewhere in the UK between 2010 and 2012. The G5 sequences from farm samples were more similar to each other than to other UK porcine rotavirus samples, but the 2014 sequences clustered separately from the 2009 and 2010 sequences.

Phylogenetic analysis of the VP4 nucleotide sequences showed that the sequences from the farm that were of the same genotype (P[6], P[7] and P[32]) were more similar to each other than to other sequences of the same genotype from elsewhere in the UK (Fig. 3b). The P[6] and P[7] sequences were identical at the amino acid level within each year of collection. The P[32] sequences found in combination with multiple VP7 genotypes were highly conserved, suggesting reassortment resulting in the same P[32] gene segment being found in combination with different VP7 genotypes, although we cannot rule out the possibility of mixed infections.

## 4 Discussion

The prevalence of rotavirus-positive faecal samples collected on the study farm was high (89% and 80% in 2009 and 2010, respectively). However, GARV are frequently isolated from asymptomatic pigs of all ages (Amimo et al., 2015, Collins et al., 2010a, Collins et al., 2010b, Lachapelle et al., 2014, Lecce and King, 1978, Steyer et al., 2008). Fu and Hampson (1987) found that only 17% of pigs shedding GARV were symptomatic.

Four rotavirus VP7 genotypes (G2, G3, G4 and G5) and three VP4 genotypes (P[6], P[7] and P[32]) were detected on the study farm and almost every combination of these genotypes was found over the 2 years. This extent of genetic heterogeneity is similar to that described in similar studies of GARV on pig farms in other countries (Amimo et al., 2013, Lachapelle et al., 2014, Miyazaki et al., 2013, Miyazaki et al., 2012).

The most common genotype combination in 2009 was G5P[32] and G4P[32] was the most common combination in 2010. In a UK-wide surveillance of porcine rotaviruses samples between 2010 and 2012 (Chandler-Bostock et al., 2014), the most common genotype combinations were G4P[6] and G5P[7]. Thus the predominant VP7 genotypes on the farm were the same as those seen in the rest of the UK, but the predominant VP4 genotypes differed. Phylogenetic analysis revealed that most VP7 sequences from the farm were more similar to each other than to sequences elsewhere in the UK, suggesting persistent transmission of viruses within the farm. The G5 sequences obtained from the farm in 2014 were related to, but distinct from, the samples isolated in 2009 and 2010, suggestive of genetic drift of isolates present on the farm, as shown in previous studies (Amimo et al., 2013, Lachapelle et al., 2014). Most of the G4 sequences from 2009 and 2010 clustered with sequences obtained from East Anglia, East Midlands and Wales in 2010 and 2011. However, one 2009 sequence and four sequences from 2014 were most similar to sequences obtained from Yorkshire and the West Midlands in 2011. The co-circulation of two variants of G4 on the farm suggests that there is some transmission of GARV between farms in England. There was less variation in the VP4 sequences, indeed the P[6] sequences obtained in 2010 and 2014 were identical at nucleotide level. Furthermore, all VP4 sequences of the same genotype collected in the same year were identical at the deduced amino acid level. The combination of an identical P[6] genotype with different VP7 genotypes (G4 and G5) between 2009 and 2010 is suggestive of reassortment, although mixed infections cannot be ruled out.

The genotyping results from 2009 and 2010 showed a significant change in the proportions of different VP7 genotypes between pre-and post-weaning samples. This shift in genotype is coincident with several factors that could potentially influence the immunological profiles of the piglets, including waning maternally-derived antibodies and the stress associated with weaning. Furthermore, in suckling pigs up to around 4 weeks of age, the predominant intestinal immunoglobulin class is IgM, with the proportion of IgA increasing post weaning, reaching about 90% at around 12 weeks of age (Allen and Porter, 1977). It has been demonstrated in various species, including pigs, that the major correlate of protection against rotavirus infection is the presence of IgA antibodies against VP4 and VP7 in the gut (Desselberger and Huppertz, 2011). That the VP4 genotypes did not differ as significantly between pre- and post-weaning samples compared to VP7 genotypes may suggest that the response against VP7 is immunodominant. However, VP4, but not VP7, interacts with glycans and host cell receptors, which may lead to greater structural constraints against variation in this protein.

It is possible that pigs exchange genotypes at weaning when they are moved from their individual farrowing rooms to a weaner pen where all the pigs weaned that week are mixed together. Alternatively, pigs may acquire a different rotavirus genotype (to which they have no prior immunity) from the new environment. Rotaviruses are known for their environmental resilience; virus particles can remain intact for over 2 years in faeces at 10 °C and in a room where no pigs had been housed for the previous 3 months (Fu and Hampson, 1987, Ramos et al., 2000). Therefore, the farm environment is undoubtedly a potential source for rotavirus infection in young pigs. A study in Japan also found by RT-PCR that pigs were continuously shedding rotavirus on farrow-to-finish farms and that changes in VP7 and VP4 genotypes were associated with movement of pigs from one building to another (Miyazaki et al., 2012). In an investigation of genetic diversity of GARV on ten finisher pig farms in Canada, Lachapelle et al., (2014) found that although strains detected in faecal samples and environmental samples were usually highly similar, on six of the farms they found different VP7 and VP4 genotypes in fomites compared to faecal samples. Taken together, these results suggest that diverse GARV strains might persist in different locations on premises, contributing to within-farm viral diversity.

## 5 Conclusions

GARV genotypes in an individual farm exhibited both temporal and spatial variation. A larger study involving a number of premises conducted over a longer period is required to determine whether there are consistent trends in within-farm variation. However, the findings are consistent with those of a Japanese study during which three to four different combinations of VP7 and VP4 genotypes were detected in samples taken during each year (over a 3-year period) and the predominant genotypes differed between pre- and post-weaning pigs and annually (Miyazaki et al., 2013). The apparent limited transmission of GARV between farms and the evidence for genetic drift suggest that different GARV strains persist within the farm environment. Changes in predominant genotypes from one year to the next may indicate that viral persistence is achieved through a combination of environmental resilience and re-emergence after naturally-acquired immunity to a particular genotype has waned, with reassortment adding to the genetic variability of the virus. These outcomes highlight the importance of strain surveillance to increase our knowledge of the molecular epidemiology of GARV, which will inform aspects of vaccine development such as the genotypes against which vaccines should afford protection.

## Figures and Tables

**Table 1:** Rotavirus genotypes identified by multiplex PCR and sequencing in (a) 2009 and (b) 2010. NT, not typed.

(a)						
VP4 genotype	VP7 genotype					Total (%)
	G2	G3	G4	G5	NT	
P[6]	4	–	5	27	31	67 (25.0%)
P[7]	–	–	–	7	5	12 (4.5%)
P[32]	34	4	9	63	51	161 (60.0%)
NT	5	–	14	9	–	28 (10.4%)

Total (%)	43 (16.0%)	4 (1.5%)	28 (10.4%)	106 (39.6%)	87 (32.5%)	268

(b)						
VP4 genotype	VP7 genotype					Total (%)
	G2	G3	G4	G5	NT	
P[6]	–	1	–	5	–	6 (1.6%)
P[7]	1	–	45	44	81	171 (44.3%)
P[32]	–	1	113	25	61	200 (51.8%)
NT	–	–	9	–	–	9 (2.3%)

Total (%)	1 (0.3%)	2 (0.5%)	167 (43.3%)	74 (19.2%)	142 (36.8%)	386

## References

[bib0005] Allen W.D., Porter P. (1977). The relative frequencies and distribution of immunoglobulin-bearing cells in the intestinal mucosa of neonatal and weaned pigs and their significance in the development of secretory immunity. Immunology.

[bib0010] Amimo J.O., Vlasova A.N., Saif L.J. (2013). Detection and genetic diversity of porcine group A rotaviruses in historic (2004) and recent (2011 and 2012) swine fecal samples in Ohio: predominance of the G9P[13] genotype in nursing piglets. J. Clin. Microbiol..

[bib0015] Amimo J.O., Junga J.O., Ogara W.O., Vlasova A.N., Njahira M.N., Maina S., Okoth E.A., Bishop R.P., Saif L.J., Djikeng A. (2015). Detection and genetic characterization of porcine group A rotaviruses in asymptomatic pigs in smallholder farms in East Africa: predominance of P[8] genotype resembling human strains. Vet. Microbiol..

[bib0020] Chandler-Bostock R., Hancox L.R., Nawaz S., Watts O., Iturriza-Gomara M., Mellits K.M. (2014). Genetic diversity of porcine group A rotavirus strains in the UK. Vet. Microbiol..

[bib0025] Collins P.J., Martella V., O’Shea H. (2008). Detection and characterization of group C rotaviruses in asymptomatic piglets in Ireland. J. Clin. Microbiol..

[bib0030] Collins P.J., Martella V., Sleator R.D., Fanning S., O’Shea H. (2010). Detection and characterisation of group A rotavirus in asymptomatic piglets in southern Ireland. Arch. Virol..

[bib0035] Collins P.J., Martella V., Buonavoglia C., O’Shea H. (2010). Identification of a G2-like porcine rotavirus bearing a novel VP4 type P[32]. Vet. Res..

[bib0040] Desselberger U., Huppertz H.I. (2011). Immune responses to rotavirus infection and vaccination and associated correlates of protection. J. Infect. Dis..

[bib0045] Fu Z.F., Hampson D.J. (1987). Group A rotavirus excretion patterns in naturally infected pigs. Res. Vet. Sci..

[bib0050] Fu Z.F., Hampson D.J. (1989). Natural transmission of group A rotavirus within a pig population. Res. Vet. Sci..

[bib0055] Gray J., Iturriza-Gomara M., Stephenson J., Warnes A. (2011). Rotaviruses. Diagnostic Virology Protocols. Methods in Molecular Biology.

[bib0060] Jeong Y.J., Matthijnssens J., Kim D.S., Kim J.Y., Alfajaro M.M., Park J.G., Hosmillo M., Son K.Y., Soliman M., Baek Y.B., Kwon J., Choi J.S., Kang M.I., Cho K.O. (2015). Genetic diversity of the VP7, VP4 and VP6 genes of Korean porcine group C rotaviruses. Vet. Microbiol..

[bib0065] Lachapelle V., Sohal J.S., Lambert M.C., Brassard J., Fravalo P., Letellier A., L’Homme Y. (2014). Genetic diversity of group A rotavirus in swine in Canada. Arch. Virol..

[bib0070] Lecce J.G., King M.W. (1978). Role of rotavirus (reo-like) in weanling diarrhea of pigs. J. Clin. Microbiol..

[bib0075] Linares R.C., Barry A.F., Alfieri A.F., Medici K.C., Grieder W., Alfieri A.A. (2009). Frequency of group A rotavirus in piglet stool samples from non-vaccinated Brazilian pig herds. Braz. Arch. Biol. Technol..

[bib0080] Martella V., Banyai K., Ciarlet M., Iturriza-Gomara M., Lorusso E., De Grazia S., Arista S., Decaro N., Elia G., Cavalli A., Corrente M., Lavazza A., Baselga R., Buonavoglia C. (2006). Relationships among porcine and human P[6] rotaviruses: Evidence that the different human P[6] lineages have originated from multiple interspecies transmission events. Virology.

[bib0085] Martella V., Ciarlet M., Bányai K., Lorusso E., Arista S., Lavazza A., Pezzotti G., Decaro N., Cavalli A., Lucente M.S., Corrente M., Elia G., Camero M., Tempesta M., Buonavoglia C. (2007). Identification of group A porcine rotavirus strains bearing a novel VP4 (P) genotype in Italian swine herds. J. Clin. Microbiol..

[bib0090] Marthaler D., Rossow K., Culhane M., Collins J., Goyal S., Ciarlet M., Matthijnssens J. (2013). Identification, phylogenetic analysis and classification of porcine group C rotavirus VP7 sequences from the United States and Canada. Virology.

[bib0095] Matthijnssens J., Ciarlet M., McDonald S.M., Attoui H., Banyai K., Brister J.R., Buesa J., Esona M.D., Estes M.K., Gentsch J.R., Iturriza-Gomara M., Johne R., Kirkwood C.D., Martella V., Mertens P.P., Nakagomi O., Parreno V., Rahman M., Ruggeri F.M., Saif L.J., Santos N., Steyer A., Taniguchi K., Patton J.T., Desselberger U., Van Ranst M. (2011). Uniformity of rotavirus strain nomenclature proposed by the Rotavirus Classification Working Group (RCWG). Arch. Virol..

[bib0100] Matthijnssens J., Otto P.H., Ciarlet M., Desselberger U., Van Ranst M., Johne R. (2012). VP6-sequence-based cut off values as a criterion for rotavirus species demarcation. Arch. Virol..

[bib0105] Miyazaki A., Kuga K., Suzuki T., Tsunemitsu H. (2012). Analysis of the excretion dynamics and genotypic characteristics of rotavirus A during the lives of pigs raised on farms for meat production. J. Clin. Microbiol..

[bib0110] Miyazaki A., Kuga K., Suzuki T., Kohmoto M., Katsuda K., Tsunemitsu H. (2013). Annual changes in predominant genotypes of rotavirus A detected in the feces of pigs in various developmental stages raised on a conventional farm. Vet. Microbiol..

[bib0115] Ramos A.P., Stefanelli C.C., Linhares R.E., de Brito B.G., Santos N., Gouvea V., de Cassia Lima R., Nozawa C. (2000). The stability of porcine rotavirus in feces. Vet. Microbiol..

[bib0120] Steyer A., Poljšak-Prijatelj M., Barlič-Maganja D., Jamnikar U., Mijovski J.Z., Marin J. (2007). Molecular characterization of a new porcine rotavirus P genotype found in an asymptomatic pig in Slovenia. Virology.

[bib0125] Steyer A., Poljsak-Prijatelj M., Barlic-Maganja D., Marin J. (2008). Human, porcine and bovine rotaviruses in Slovenia: evidence of interspecies transmission and genome reassortment. J. Gen. Virol..

[bib0130] Trojnar E., Sachsenroder J., Twardziok S., Reetz J., Otto P.H., Johne R. (2013). Identification of an avian group A rotavirus containing a novel VP4 gene with a close relationship to those of mammalian rotaviruses. J. Gen. Virol..

[bib0135] Zhou X., Wang Y.H., Souvik G., Tang W.F., Pang B.B., Liu M.Q., Peng J.S., Zhou D.J., Kobayashi N. (2015). Genomic characterization of G3P[6], G4P[6] and G4P[8] human rotaviruses from Wuhan, China: evidence for interspecies transmission and reassortment events. Infect. Genet. Evol..

